# Estimation of anaerobic threshold by cardiac repolarization instability: a prospective validation study

**DOI:** 10.1186/s13102-021-00312-1

**Published:** 2021-08-06

**Authors:** Dominik Schüttler, Simone Krammer, Lukas von Stülpnagel, Lauren Sams, Axel Bauer, Wolfgang Hamm, Stefan Brunner

**Affiliations:** 1 Department of Medicine I, University Hospital Munich, Campus Grosshadern and Innenstadt, Ludwig-Maximilians University Munich (LMU), Ziemssenstrasse 1, 80336 Munich, Germany; 2 DZHK (German Centre for Cardiovascular Research), Partner Site Munich, Munich Heart Alliance (MHA), Munich, Germany; 3 Ludwig-Maximilians University Munich (LMU), Walter Brendel Centre of Experimental Medicine, Munich, Germany; 4 Medical University Innsbruck, University Hospital for Internal Medicine III, Innsbruck, Austria

**Keywords:** Cardiac repolarization, Autonomic nervous system, Anaerobic threshold, Professional athlete

## Abstract

**Background:**

Assessing lactate (LT) or anaerobic thresholds (AT) in athletes is an important tool to control training intensities and to estimate individual performance levels. Previously we demonstrated that ECG-based assessment of cardiac repolarization instability during exercise testing allows non-invasive estimation of AT in recreational athletes. Here, we validate this method in professional and amateur team sports athletes.

**Methods:**

We included 65 team sports athletes (32 professionals and 33 amateur athletes; 51 men, 14 women, mean age 22.3 ± 5.2 years) undergoing a standardized incremental cycle exercise test. During exercise testing a high-resolution ECG (1000 Hz) was recorded in Frank-leads configuration and beat-to-beat vector changes of cardiac repolarization (dT°) were assessed by previously established technologies. Repolarization-based AT (AT_dT°_) was estimated by its typical dT°-signal pattern. Additionally, LT was detected in accordance to methods established by Mader (LT_Mader_) and Dickhuth (LT_Dickhuth_).

**Results:**

All athletes performed exercise testing until exhaustion with a mean maximum workload of 262.3 ± 60.8 W (241.8 ± 64.4 W for amateur athletes and 283.4 ± 49.5 W for professional athletes). Athletes showed AT_dT°_ at 187.6 ± 44.4 W, LT_Dickhuth_ at 181.1 ± 45.6 W and LT_Mader_ at 184.3 ± 52.4 W. AT_dT°_ correlated highly significantly with LT_Dickhuth_ (*r* = 0.96, *p* < 0.001) and LT_Mader_ (*r* = 0.98, *p* < 0.001) in the entire cohort of athletes as well as in the subgroups of professional and amateur athletes (*p* < 0.001 for all).

**Conclusions:**

AT_dT°_, defined by the maximal discordance between dT° and heart rate, can be assessed reliably and non-invasively via the use of a high-resolution ECG in professional and amateur athletes.

## Background

Controlling exercise intensities has been shown to be one of the key measures to improve endurance capacity and performance: The concept of using sub-maximal workload parameters such as lactate (LT) or ventilatory thresholds (VT) to determine individual cardio-respiratory fitness and to schedule training intensities is commonly accepted in this context and has been demonstrated in endurance as well as team-sports athletes [1–3].

Assessment of parameters of the autonomic nervous system (ANS) has gained pronounced attention. The concept of testing biomarkers of the ANS for estimating thresholds is based on the idea that the ANS exerts distinct influences on the cardiorespiratory system during exercise to regulate heart rate, cardiac contractility and blood pressure. Recently, different study groups implemented the evaluation of autonomic biomarkers to control training intensities and to detect training-induced states of fatigue [4]. Measuring ANS biomarkers is promising as it is non-invasive and cost-efficient. Heart rate variability (HRV)-derived parameters reflecting ANS activity showed an association with LT in healthy and diseased cohorts [5–8]. Despite different methods to assess AT including metabolic markers (lactate, glucose), ventilatory responses, autonomic markers (e.g. HRV-derived markers such as RMSSD) or neuroendocrine markers (catecholamines), they all determine AT within close ranges [9]. However, as the physiological downstream is faster in some systems than in others, these different methods assessing the anaerobic threshold may show slightly different workloads at the AT. Nevertheless, as all these methods differ in invasiveness, costs, time-consumption and easiness of determination, new methods to indicate AT may be helpful.

Sympathetic-activity associated periodic repolarization dynamics (PRD) is a novel ECG-based parameter, that reflects influences of efferent cardiac sympathetic activity on the ventricular myocardium during repolarization [10, 11]. The non-invasive assessment via high-resolution ECG is based on beat-to-beat changes of the T wave vector (dT°) with periodic components of repolarization in the low-frequency range (≤ 0.1 Hz). Large clinical trials demonstrated that increased levels of PRD are strong predictors of sudden cardiac death (SCD) in patients with ischemic and non-ischemic cardiomyopathy [11–13].

As exercise is known to affect ventricular repolarization crucially [4, 14] we previously tested repolarization instability (dT° signal) during exercise testing [15]: We demonstrated that the dT° signal shows a characteristic three-phasic pattern that allows a reliable and non-invasive estimation of the anaerobic threshold (we called it AT_dT°_) in healthy recreational athletes. This pattern at the anaerobic threshold is characterized by a maximal discordance of dT° and heart rate and this point highly significantly correlated with lactate thresholds measured by the methods of Mader and Dickhuth [15].

In the present study we validated this non-invasive ECG-based assessment of the anaerobic threshold by our previously described methods in a cohort of 65 team sport athletes to check if this method can be transferred to professional athletes as well as well-trained amateur athletes who are able to achieve markedly higher maximal workloads during exercise and have increased workloads at lactate thresholds.

## Methods

### Study population

We included 65 healthy team sport athletes (14 women, 51 men, mean age 22.3 ± 5.2 year. (standard deviation), minimum age 14 year., maximum age 36 year.) who underwent a graded cycle ergometer test until maximal exhaustion. Our study cohort consisted of 32 professional athletes (1st league and 2nd league European football clubs and 1st league basketball club) and 33 amateur athletes (4th and 6th league European football clubs). Exclusion criteria were acute or chronic infections, presence of pacemakers or implantable cardioverter defibrillators (ICDs), history of cardiovascular diseases or risk factors and other contraindications for performing exercise testing [16].

All individuals gave written informed consent. For participants under 16 years old, written informed consent was obtained from a parent or guardian. This study was approved by the local ethics committee (*Ethikkommission der Medizinischen Fakultät der LMU München*) and was conducted in accordance to the Declaration of Helsinki.

### Exercise testing

All subjects performed a standardized graded cycle exercise test [17] (starting at 90 W workload) until fatigue which we defined as not being capable to maintain pedal cadence above 70 rpm. Increases of 30 W took place every 3 min while pedal cadence was kept constantly at 70–90 rpm.

### Assessment of ECG-based cardiac repolarization instability and detection of anaerobic threshold via dT° (AT_(dT°)_)

**Fig. 1:**
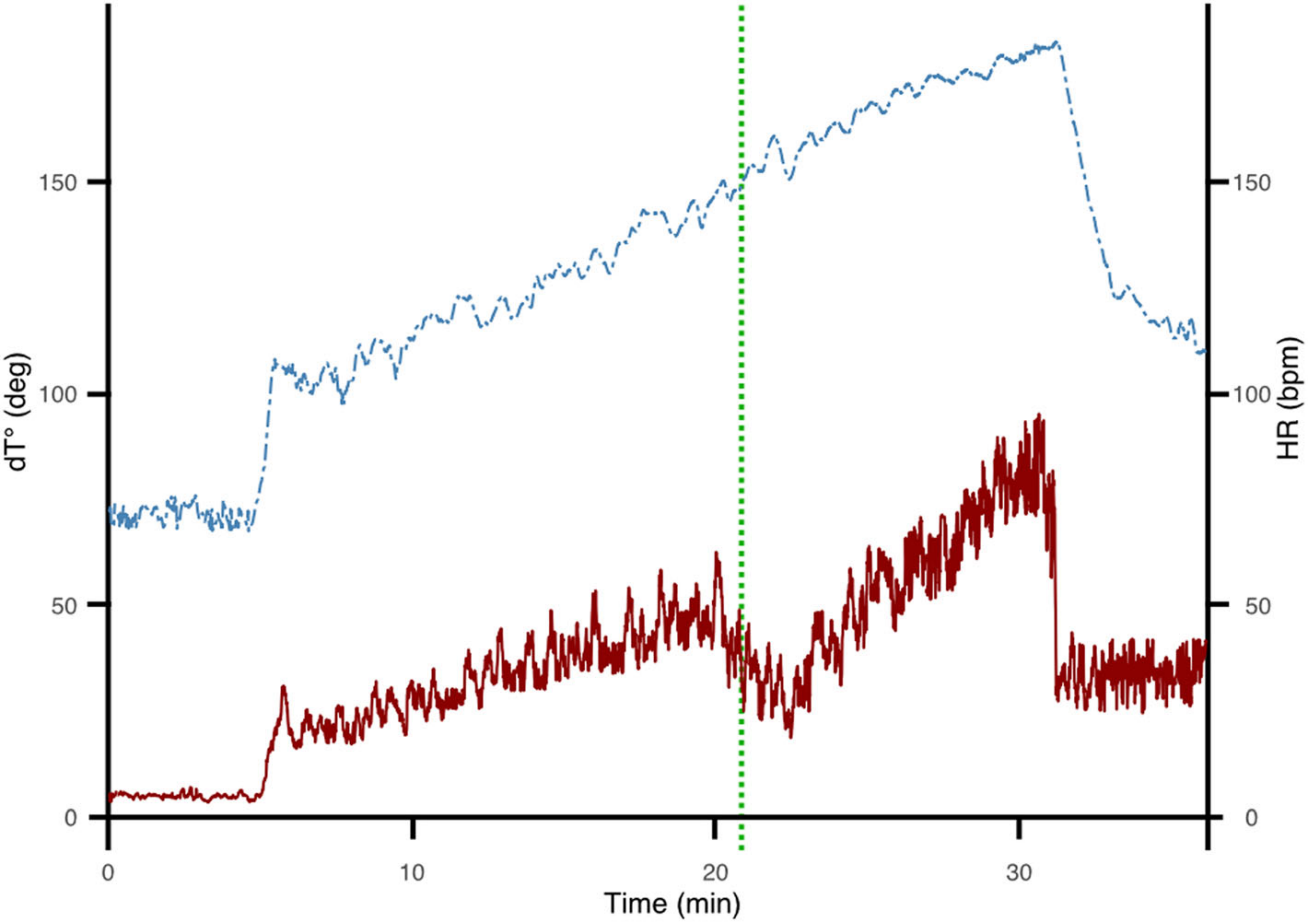
Exemplary dT° signal (red line) and corresponding heart rate (blue line) in the course of time during a graded exercise test. Green dotted line indicates the moment of minimal concordance of heart rate and dT° defined as AT_dT°_

Determination of AT via dT° signals was carried out analogously to a previous study by our group [15]. For details, we thus refer to this publication. In brief, we analyzed high-resolution data from Frank’s orthogonal lead ECG (1000 Hz, Schiller medilog AR4 plus, Schiller diagnostics, CH) which was recorded throughout the entire exercise test including a 5 min resting phase prior and after the end of each cycle test with SMARTlab computer and R peak and T wave detection algorithms [17, 18]. In this process, the obtained spatiotemporal properties of each T wave are then used to assess the angle dT° between two successive repolarization (T wave) vectors [11, 13, 15]. When plotted over time dT° displays a variability with typical underlying oscillations in the low-frequency range (≤ 0.1 Hz) [11, 13, 15]. During exercise this dT° signal shows a characteristic three-phasic pattern and AT_dT°_ is defined as the point of maximal discordance between dT° signal and heart rate [15]. Figure 1 illustrates an exemplary dT° signal and corresponding heart rate signal during graded exercise test and shows the moment of maximal discordance between these signals defined as AT_dT°_. This point was converted into power output (W) assuming a linear increase in exercise increments and the corresponding heart rate was extracted from the ECG signal.

### Detection of lactate thresholds via methods by Mader and Dickhuth

Capillary blood samples were obtained from earlobes and lactate concentrations (in mmol/l) were measured (lactate Scout+, EKF Diagnostics, Cardiff, GB) before exercise at rest, during exercise at the end of each incremental step and after exercise. The individual lactate threshold was calculated using a standardized computer software (winlactat V 5.2.1.6., Mesics, Münster, Germany). The calculation of LT was determined according to the methods by Mader (fixed threshold at 4 mmol/l) and Dickhuth [1, 19]. Dickhuth definded the LT as the lactate concentration 1.5 mmol/l above the lactate equivalent (i.e. the lowest value of the lactate-performance ratio marking the onset of the lactate increase during exercise) [19].

### Statistics

All results are represented as mean ± standard deviation. For statistical analyses and graphical illustration, we used CRAN “R” 3.6.3. Kruskall-Wallis test was performed to detect statistical differences between mean determined thresholds (AT_dT°_, LT_Mader_ and LT_Dickhuth_). The relationship between the three different methods was assessed using the Pearson correlation coefficient test. Intra class correlation (ICC) was tested for these three methods. Bland-Altman plots were performed to visualize the differences between the methods and the respective average.

## Results

### Baseline characteristics of study participants

Table 1 shows the baseline characteristics of all study participants (A) as well as of professional athletes (B) and amateur athletes (C) as subgroups. Altogether 65 healthy team sport athletes (14 women, 51 men, mean age 22.3 ± 5.2 yrs.) were included in this study. Mean BMI was 22.7 ± 1.8 kg×m^− 2^, mean maximum workload was 262.3 ± 60.8 W (241.8 ± 64.4 W for amateur athletes and 283.4 ± 49.5 W for professional athletes). All participants finished exercise testing until exhaustion. Table 2 shows performance parameters, heart rates and lactate thresholds assessed via methods by Mader and Dickhuth as well as AT_dT°_ for all participants (A) and professional (B) and amateur athletes (C) alone. To exclude that maximal performance was not reached during the test, we calculated theoretical maximal heart rate using the formula 208-(age x 0.7) as described elsewhere [20] and calculated %HR_max expected_ for all thresholds.

**Table 1:** Shows baseline characteristics for all participants (A) and subgroups of professional athletes and amateur athletes. All data presented as mean ± standard deviation. *BMI *body mass index

	(A) all participants (*n* = 65)	(B) professional athletes (*n* = 32)	(C) amateur athletes (*n* = 33)
female (n)	14	0	14
male (n)	51	32	19
age (yr)	22.3 ± 5.2	23.1 ± 5.5	21.6 ± 4.9
weight (kg)	73.9 ± 10.5	78.5 ± 8.4	69.4 ± 10.5
height (cm)	180.2 ± 9.6	185.4 ± 7.5	175.1 ± 8.6
BMI (kg×m^− 2^)	22.7 ± 1.8	22.8 ± 1.3	22.5 ± 2.1

**Table 2:** Shows parameters of performance and heart rates in means ± standard deviation. LTs calculated via method by Mader and Dickhuth and AT assessed via dT°. *HR *heart rate, *bpm *beats per minute, *PO *power output, *W *Watt, *LT *lactate threshold, *AT *anaerobic threshold. Maximal expected heart rate calculated using the formula HR_max(expected)_(bpm) = 208-(age x 0.7)

	(A) all participants (*n* = 65)	(B) professional athletes (*n* = 32)	(C) amateur athletes (*n* = 33)
PO_max_ (W)	262.3 ± 60.8	283.4 ± 49.5	241.8 ± 64.4
LT_Dickhuth_: PO (W)	181.1 ± 45.6	196.2 ± 37.0	166.5 ± 48.8
LT_Dickhuth_: %PO_max_	69.0 ± 6.4	69.2 ± 5.6	68.9 ± 7.2
LT_Mader_: PO (W)	184.3 ± 52.4	198.6 ± 41.5	170.4 ± 58.6
LT_Mader_: %PO_max_	69.7 ± 8.7	70.0 ± 8.0	69.5 ± 9.5
AT_dT°_: PO (W)	187.6 ± 44.4	204.1 ± 33.9	171.7 ± 47.9
AT_dT°_:%PO_max_	71.8 ± 6.8	72.3 ± 5.2	71.3 ± 8.0
LT_Dickhuth_: HR (bpm)	152.8 ± 14.9	151.0 ± 15.7	154.3 ± 14.4
LT_Mader_: HR (bpm)	153.1 ± 14.9	151.3 ± 15.8	154.6 ± 14.1
AT_dT°_: HR (bpm)	154.0 ± 14.2	151.7 ± 14.3	156.0 ± 14.1
HR_max(expected)_(bpm)	192.4 ± 3.7	191.9 ± 3.8	192.9 ± 3.5
LT_Dickhuth_: % HR_max(expected)_	79.4 ± 7.2	78.7 ± 7.3	80.0 ± 7.2
LT_Mader_: % HR_max(expected)_	79.6 ± 7.3	78.9 ± 7.4	80.2 ± 7.3
AT_dT°_: % HR_max(expected)_	80.0 ± 6.9	79.1 ± 6.6	80.9 ± 7.1

### dT° signal pattern during exercise and determination of AT_(dT°)_

We were able to identify the typical, previously described three-phasic dT° pattern [15] in all participating athletes during cycle exercise test: We found a low dT° signal at rest (first 15 min) which immediately increased concordantly to the heart rate with the beginning of exercising. At AT_dT°_, dT° and heart rate showed minimal correlation. Then dT° transiently declines before increasing again until the end of the exercise test (exemplary signal: see Fig. 1). During recovery the dT° signal drops but remains higher than baseline levels. AT_dT°_ was determined as previously described [15].

### Correlation of AT_dT°_ with LTs by Mader and Dickhuth

AT_dT°_ occurred at 187.6 ± 44.4 W, LT_Dickhuth_ at 181.1 ± 45.6 W and LT_Mader_ at 184.3 ± 52.4 W. Mean heart rate at AT_dT°_ was 154.0 ± 14.2 bpm, at LT_Dickhuth_ 152.8 ± 14.9 bpm and at LT_Mader_ 153.1 ± 14.9 bpm (Table 2).

**Fig. 2:**
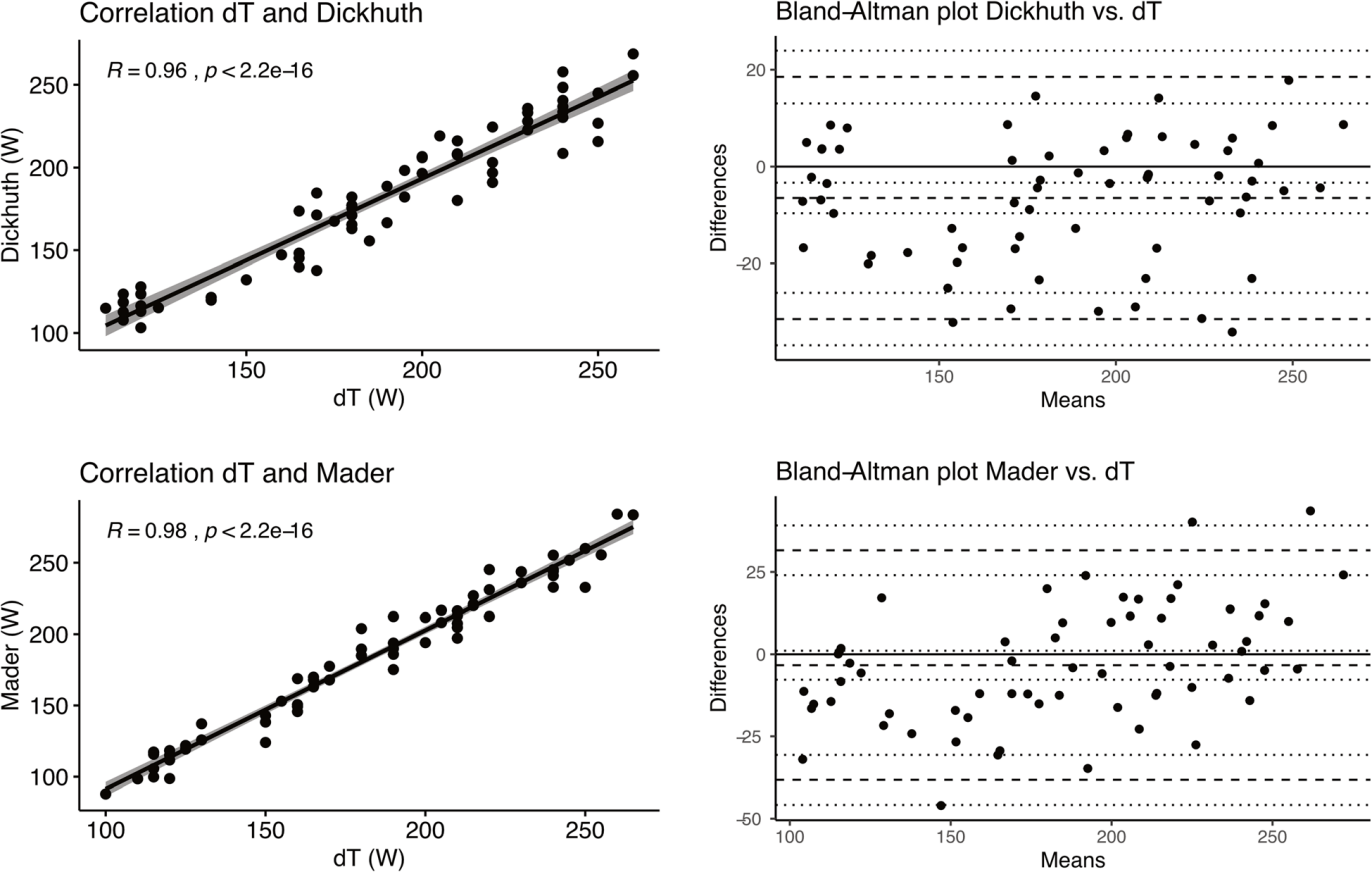
Pearson correlation coefficient test for power output between AT_dT°_ and LT_Dickhuth_/LT_Mader_. Bland-Altman plots illustrate concordance between AT_dT°_ and LT_Dickhuth_ as well as between AT_dT°_ and LT_Mader_

AT_dT°_ highly significantly correlated with LT_Dickhuth_ (*R* = 0.96, R^2^ = 0.92, *p* < 0.001) and LT_Mader_ (*R* = 0.98, R^2^ = 0.96, *p* < 0.001) (Fig. 2A and C, respectively) investigating power output. Similar results were detectable correlating heart rates at AT_dT°_ with LT_Dickhuth_ (*R* = 0.97, R^2^ = 0.94, *p* < 0.001) and with LT_Mader_ (*R* = 0.92, R^2^ = 0.85, *p* < 0.001). Intra class correlation for these three methods was excellent with intraclass correlation coefficients (ICC) of 0.95 (power output) and 0.93 (heart rate). Bland-Altman plots illustrate close concordance between AT_dT°_ and LT_Dickhuth_ (Fig. 2B) as well as between AT_dT°_ and LT_Mader_ (Fig. 2D) with power output showing a mean difference of 6.6 W between AT_dT°_ and LT_Dickhuth_, of 3.3 W between AT_dT°_ and LT_Mader_ and of 3.2 W between LT_Dickhuth_ and LT_Mader_.

**Fig. 3:**
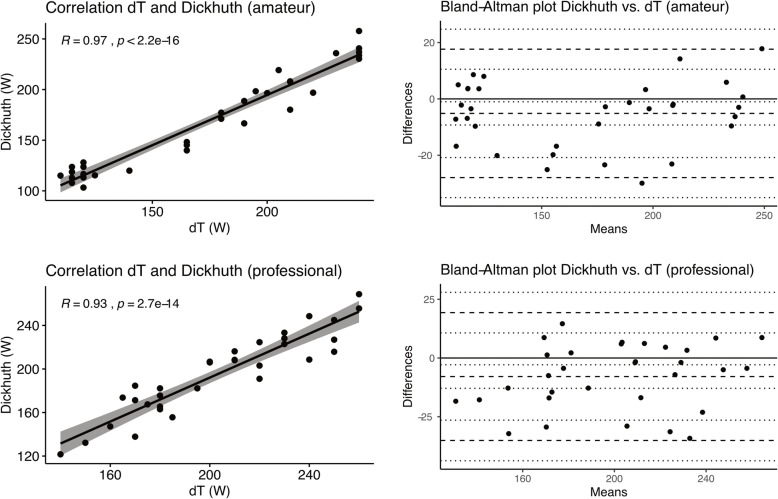
Pearson correlation coefficient test for power output between AT_dT°_ and LT_Dickhuth_ for amateur athletes (**A**) and professional athletes (**C**). Bland-Altman plots indicate concordance between AT_dT°_ and LT_Dickhuth_ (**B** + **D**)

**Fig. 4:**
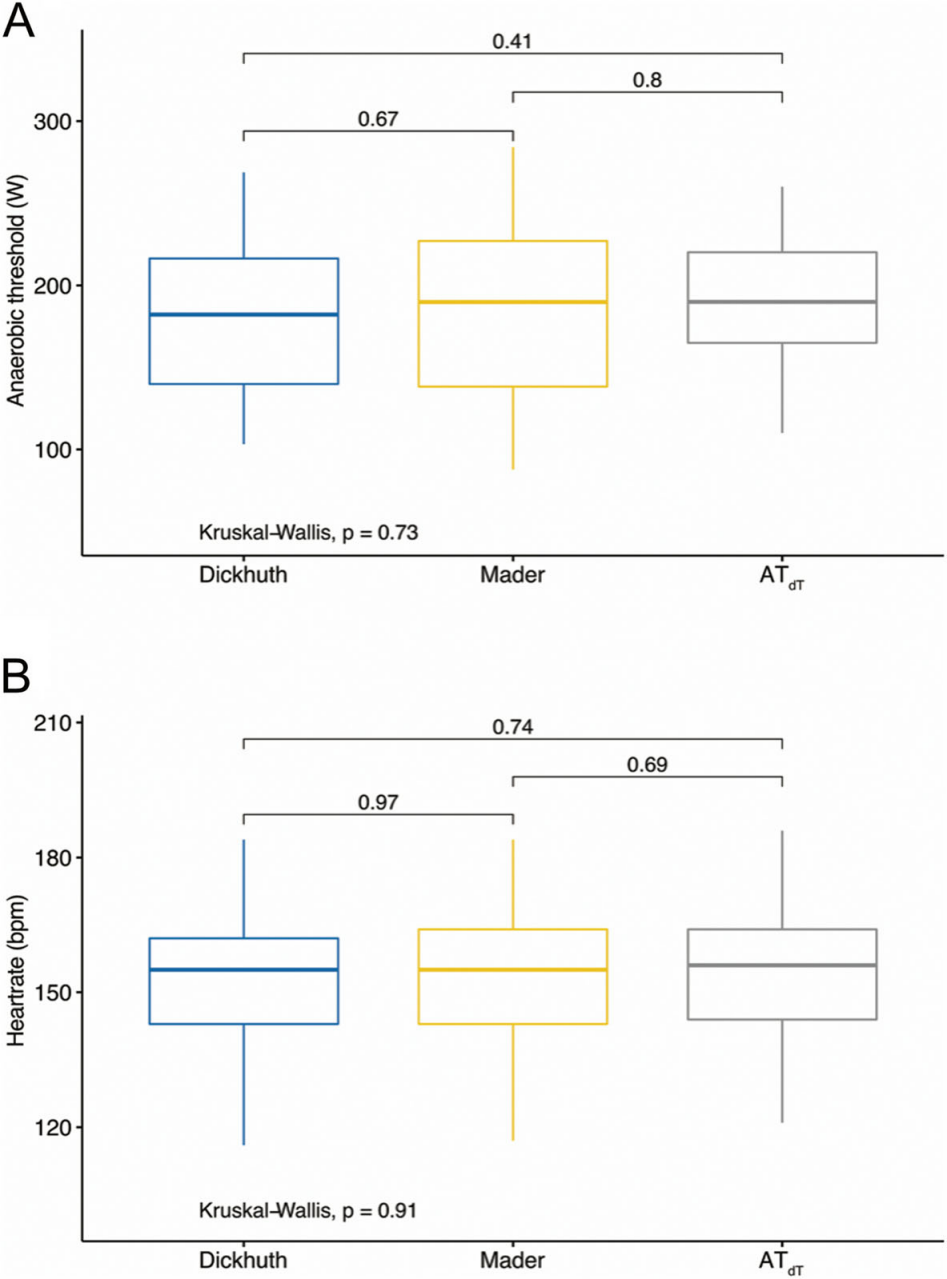
Box plots for power output (**A**) and heart rate (**B**) at AT_dT°_, LT_Mader_ and LT_Dickhuth_. Kruskal-Wallis test shows no statistical differences between performed measurements to detect AT

We further investigated thresholds for subgroups of professional athletes (*n* = 32) and amateur athletes (*n* = 33). In professional athletes mean AT_dT°_ was at 204.1 ± 33.9 W, LT_Dickhuth_ at 196.2 ± 37.0 W and LT_Mader_ at 196.2 ± 37.0 W (ICC = 0.91). Elite athletes had a mean heart rate of 151.7 ± 14.3 bpm at AT_dT°_ of 151.0 ± 15.7 bpm at LT_Dickhuth_ and of 151.3 ± 15.8 bpm at LT_Mader_ (ICC = 0.94). In amateur athletes we detected AT_dT°_ at 171.7 ± 47.9 W, LT_Dickhuth_ at 166.5 ± 48.8 W and LT_Mader_ at 170.4 ± 58.6 W (ICC = 0.96). Amateur athletes had a mean heart rate of 156.0 ± 14.1 bpm at AT_dT°_ of 154.3 ± 14.4 at LT_Dickhuth_ and of 154.6 ± 14.1 at LT_Mader_ (ICC = 0.93). Figure 3 visualizes the strong correlation between AT_dT°_ and LT_Dickhuth_ for both subgroups of amateur (*R* = 0.97, R^2^ = 0.94, *p* < 0.001, Fig. 3A and B) and professional athletes (*R* = 0.93, R^2^ = 0.86, *p* < 0.001, Fig. 3C and D) regarding power output. Figure 4A shows box plots for power outputs at AT_dT°_, LT_Mader_ and LT_Dickhuth_ with no significant differences between methods of threshold determination. Intergroup comparison revealed no significant differences as checked by Kruskal-Wallis test (*p* = 0.73). Figure 4B shows box plots for heart rates at AT_dT°_, LT_Mader_ and LT_Dickhuth_ with no significant differences between methods of threshold determination. Kruskal-Wallis test again detected no significant differences between assessment methods (*p* = 0.91).

## Discussion

In the present study we were able to validate a non-invasive cardiac repolarization-based method [15] to determine AT in a large cohort of professional and amateur team sport athletes while performing a standardized incremental cycle exercise test. We confirmed the characteristic three-phasic pattern in all study participants who showed a gradual increase concordantly to the heart rate at the start of exercise, a sudden drop in repolarization instability (here at a power output of mean 187.6 ± 44.4 W) discordantly to the heart rate and a new rise of the dT° signal at the end of the workout. The moment of minimal concordance of dT° and heart rate (AT_dT°_) correlated highly significantly with lactate thresholds by Mader and Dickhuth.

Therefore, this pattern has been shown to be characteristic in both young and healthy average-trained athletes as well as professional athletes and very well-trained amateur athletes undergoing standardized incremental cycle exercise tests. It is thus valid and reproducible in both athletes who are only able to perform exercise until lower maximal workloads and in athletes capable to continue exercise tests up to very high maximum workloads. This determination of AT is similar to results previously published by Milagro et al. who also found changes in the profile of ventricular repolarization instability as well as of oscillations in the low frequency spectrum once reaching the anaerobic threshold [21]. It is noteworthy that these changes in repolarization instability cannot be provoked by increased heart rate or by fixed atrial pacing [11, 15]. The dT° signal occurs independently of breathing rates and is not associated with heart rate variability [11]. Emerging data validly attributes the dT° signal to efferent sympathetic cardiac nerve activity at the ventricular myocardium [11, 22].

Studies have broadly investigated the alterations within the autonomic nervous system during exercise and the mechanisms found are dynamic, complex and still remain incompletely understood: It is established that the activity of the sympathetic ANS gradually increases with greater workloads shifting from an approximately 4:1 vagal-sympathetic balance to a 4:1 sympatho-vagal balance in the course of an exercise [23]. The change in autonomic cardiac modulation during increasing workload subsequently results in altered HRV-derived parameters and this has been used to assess AT in athletes [24, 25]. Our present study, studies assessing AT via HRV-derived parameters [24, 25] and previous studies investigating the connection of changes in repolarization instability and AT [15, 21] thus suggest that the autonomic control of the electrical activity of the myocardium, especially on the level of the ventricular myocardium seems to change once reaching the anaerobic threshold.

While the exact physiological mechanisms underlying AT_dT°_ remain unclear, our study provides several relevant practical implications for future sports research and training physiology: (i) We were able to present a method which is non-invasive and does not require any puncture to gain blood samples. (ii) Furthermore, as it is ECG-based it is less cost-intensive as point-of-care lactate measurements. (iii) A link between HRV indices and blood lactate levels was found during resistance exercise [6–8], in endurance athletes [5] and team sports athletes [24, 25]. Of note, HRV indices are dynamic and rather sensitive to environmental conditions [4]. dT° was proven to be independent of heart rate and breathing rate [11]. Nevertheless, up to date there has no study been conducted comparing the assessment of thresholds based on HRV indices with repolarization patterns. (iv) As threshold-based training has been shown to lead to performance benefits in endurance as well as team-sports athletes [1–3] the investigation of AT_dT°_-based training intensities in athletes might be of high interest and should be investigated in future studies.

Our study has some limitations. First, we investigated only team sport athletes and the group of professional athletes contained no female athletes. Whether determination of LTs via AT_dT°_ can be validly transferred to endurance athletes has to be elucidated in future studies. Secondly, we only investigated the dT° signal on a cycle ergometer and not with different methods such as treadmill running. Third, AT_dT°_ was only correlated to LT and not to ventilatory thresholds as assessed during cardiopulmonary exercise testing. This should be performed in future studies. Additionally, we performed graded exercise tests. However, the gold standard to determine lactate thresholds is the assessment of the maximal steady state during multiple rectangular exercise protocols. Finally, our study included healthy individuals without any known underlying cardiovascular diseases. Whether the assessment of AT_dT°_ can be performed in patients with cardiovascular disease or on medication influencing the ANS has to be investigated in future studies.

## Conclusions

We demonstrated that AT_dT°_ is a reliable and non-invasive measurement to assess AT. It correlates with established methods of LT assessment in a large cohort of professional and well-trained amateur athletes. The results of this validation study indicate that AT_dT°_ might represent a promising tool for future routine application.

## Data Availability

All data can be obtained by request from the corresponding author.

## Abbreviations

ANS: Autonomic nervous system
AT: Anaerobic threshold
BMI: Body mass index
ECG: Electrocardiogram
HRV: Heart rate variability
ICD: Implantable cardioverter defibrillator
LT: Lactate threshold
PRD: Periodic repolarization dynamics
SCD: Sudden cardiac death
VT: Ventilatory threshold

## References

[1] Faude O, Kindermann W, Meyer T (2009). Lactate threshold concepts: how valid are they?. Sports Med.

[2] Manzi V, Bovenzi A, Franco Impellizzeri M, Carminati I, Castagna C (2013). Individual training-load and aerobic-fitness variables in premiership soccer players during the precompetitive season. J Strength Cond Res.

[3] Pyne DB, Lee H, Swanwick KM (2001). Monitoring the lactate threshold in world-ranked swimmers. Med Sci Sports Exerc.

[4] Schüttler D, Hamm W, Bauer A, Brunner S (2020). Routine heart rate-based and novel ECG-based biomarkers of autonomic nervous system in sports medicine. Dtsch Z Sportmed.

[5] Di Michele R, Gatta G, Di Leo A, Cortesi M, Andina F, Tam E (2012). Estimation of the anaerobic threshold from heart rate variability in an incremental swimming test. J Strength Cond Res.

[6] Simoes RP, Castello-Simoes V, Mendes RG, Archiza B, Dos Santos DA, Bonjorno JC (2014). Identification of anaerobic threshold by analysis of heart rate variability during discontinuous dynamic and resistance exercise protocols in healthy older men. Clin Physiol Funct Imaging.

[7] Simoes RP, Mendes RG, Castello V, Machado HG, Almeida LB, Baldissera V (2010). Heart-rate variability and blood-lactate threshold interaction during progressive resistance exercise in healthy older men. J Strength Cond Res.

[8] Simoes RP, Mendes RG, Castello-Simoes V, Catai AM, Arena R, Borghi-Silva A (2016). Use of Heart Rate Variability to Estimate Lactate Threshold in Coronary Artery Disease Patients during Resistance Exercise. J Sports Sci Med.

[9] Sales MM, Sousa CV, da Silva Aguiar S, Knechtle B, Nikolaidis PT, Alves PM (2019). An integrative perspective of the anaerobic threshold. Physiol Behav.

[10] Hamm W, Maier F, Kassem S, Schuttler D, Bauer A, Rizas KD (2020). Deceleration capacity of heart rate and periodic repolarization dynamics during normobaric hypoxia. Scand J Med Sci Sports.

[11] Rizas KD, Nieminen T, Barthel P, Zurn CS, Kahonen M, Viik J (2014). Sympathetic activity-associated periodic repolarization dynamics predict mortality following myocardial infarction. J Clin Invest.

[12] Bauer A, Klemm M, Rizas KD, Hamm W, von Stulpnagel L, Dommasch M (2019). Prediction of mortality benefit based on periodic repolarisation dynamics in patients undergoing prophylactic implantation of a defibrillator: a prospective, controlled, multicentre cohort study. Lancet.

[13] Rizas KD, McNitt S, Hamm W, Massberg S, Kaab S, Zareba W (2017). Prediction of sudden and non-sudden cardiac death in post-infarction patients with reduced left ventricular ejection fraction by periodic repolarization dynamics: MADIT-II substudy. Eur Heart J.

[14] Ali A, Mehra MR, Malik FS, Lavie CJ, Bass D, Milani RV (1999). Effects of aerobic exercise training on indices of ventricular repolarization in patients with chronic heart failure. Chest.

[15] Hamm W, von Stülpnagel L, Rizas KD, Vdovin N, Klemm M, Bauer A (2019). Dynamic Changes of Cardiac Repolarization Instability during Exercise Testing. Med Sci Sports Exerc.

[16] Lollgen H, Leyk D (2018). Exercise Testing in Sports Medicine. Dtsch Arztebl Int.

[17] Jameson C, Ring C (2000). Contributions of local and central sensations to the perception of exertion during cycling: effects of work rate and cadence. J Sports Sci.

[18] Laguna P, Jane R, Caminal P (1994). Automatic detection of wave boundaries in multilead ECG signals: validation with the CSE database. Comput Biomed Res.

[19] Dickhuth HH, Yin L, Niess A, Rocker K, Mayer F, Heitkamp HC (1999). Ventilatory, lactate-derived and catecholamine thresholds during incremental treadmill running: relationship and reproducibility. Int J Sports Med.

[20] Roy S, McCrory J (2015). Validation of Maximal Heart Rate Prediction Equations Based on Sex and Physical Activity Status. Int J Exerc Sci.

[21] Milagro J, Hernandez-Vicente A, Hernando D, Casajus JA, Garatachea N, Bailon R (2021). Estimation of the second ventilatory threshold through ventricular repolarization profile analysis. Scand J Med Sci Sports.

[22] Hanson B, Child N, Van Duijvenboden S, Orini M, Chen Z, Coronel R (2014). Oscillatory behavior of ventricular action potential duration in heart failure patients at respiratory rate and low frequency. Front Physiol.

[23] White DW, Raven PB (2014). Autonomic neural control of heart rate during dynamic exercise: revisited. J Physiol.

[24] Cottin F, Medigue C, Lopes P, Lepretre PM, Heubert R, Billat V (2007). Ventilatory thresholds assessment from heart rate variability during an incremental exhaustive running test. Int J Sports Med.

[25] Ramos-Campo DJ, Rubio-Arias JA, Avila-Gandia V, Marin-Pagan C, Luque A, Alcaraz PE (2017). Heart rate variability to assess ventilatory thresholds in professional basketball players. J Sport Health Sci.

